# Clinical and Market Analysis of NanoBEO: A Public-Worth, Innovative Therapy for Behavioral and Psychological Symptoms of Dementia (BPSD)—Emerging Evidence and Its Implications for a Health Technology Assessment (HTA) and Decision-Making in National Health Systems

**DOI:** 10.3390/pharmaceutics16101253

**Published:** 2024-09-27

**Authors:** Damiana Scuteri, Daniele Pierobon, Martina Pagliaro, Kengo Hamamura, Takafumi Hayashi, Loris Pignolo, Pierluigi Nicotera, Giacinto Bagetta, Maria Tiziana Corasaniti

**Affiliations:** 1 Department of Health Sciences, University “Magna Graecia” of Catanzaro, 88100 Catanzaro, Italy; mtcorasa@unicz.it; 2 Consultant for Knowledge Valorization and Technology Transfer of Life Science Projects, 10024 Torino, Italy; dpierobo@gmail.com; 3 Preclinical and Translational Pharmacology, Department of Pharmacy, Health Science and Nutrition, University of Calabria, 87036 Cosenza, Italy; martina.pagliaro@unical.it (M.P.); g.bagetta@unical.it (G.B.); 4 Department of Clinical Pharmacokinetics, Faculty of Pharmaceutical Sciences, Kyushu Univerity, 3-1-1 Maidashi Higashi-ku, Fukuoka 812-8582, Japan; hamamura@phar.kyushu-u.ac.jp; 5 Division of Pharmaceutics, Faculty of Pharmaceutical Sciences, Tohoku Medical and Pharmaceutical University, Sendai 981-8558, Japan; thayashi@tohoku-mpu.ac.jp; 6 Regional Center for Serious Brain Injuries, S. Anna Institute, 88900 Crotone, Italy; l.pignolo@isakr.it; 7 The German Center for Neurodegenerative Diseases (DZNE), 53127 Bonn, Germany; pierluigi.nicotera@dzne.de

**Keywords:** NanoBEO, HTA, clinical and market analysis, essential oil of bergamot, dementia, BPSD, agitation, pain, nanotechnology delivery system

## Abstract

Background: According to scientific literature, some 99% of patients affected by Alzheimer’s disease (AD) suffer from behavioral and psychological symptoms of dementia (BPSD), also known as neuropsychiatric symptoms (NPSs). In particular, agitation is one of the most difficult disorders to treat. States of agitation represent a very serious problem as they make these subjects dangerous for themselves and others and worsen as the disease advances. To date, there are no specific solutions for treating agitation. The only authorized drug is risperidone (as well as brexpiprazole, approved by the FDA on 11 May 2023), which can be used for no longer than 6–12 weeks because it increases the risk of death—owing to cardiocerebrovascular accidents—by 1.6–1.7 times. Methods: In order to address the latter noteworthy unmet medical need, NanoBEO was produced. The aim of the present work is to generate the health technology assessment (HTA) of this nanotechnological device. The latter consists of a controlled release system, based on solid lipid nanoparticles loaded with bergamot essential oil (BEO). Results: The results of the present research assessed the current evidence in the field of non-pharmacological treatments for this condition, including relevant primary preclinical and clinical data studies supporting the use of this device and the production of the operative plan for its launch on the market. The findings offer recommendations for decision-making on its implementation in dementia. Conclusions: NanoBEO represents a public-worth innovation in this neglected area, marking a significant advancement in the history of dementia, moving from academic research to product development.

## 1. Introduction

The increase in life expectancy due to modern medicine and improved standards of living during the 21st century has led to a demographic aging process that tackles age-related conditions [1], with a constant increase in age-related neurodegenerative diseases, despite the recent pandemic (during which, those diseases were fundamental risk factors) [2]. Dementia is a public health priority, with approximately 55 million people affected worldwide and approximately 41 million remaining undiagnosed [3]. It is estimated that 75 million people will be affected by 2030 and 140 million by 2050 [4]. Among the different forms of dementia, Alzheimer’s disease (AD) is the most frequent condition, accounting for approximately two-thirds of all cases [5,6]. However, other forms of dementia include Lewy body dementia; frontotemporal dementia; and vascular dementia. The World Health Organization (WHO) defines dementia as a clinical syndrome resulting from brain pathology of a progressive nature, leading to disturbances of multiple higher cortical functions, including memory, thinking, orientation, understanding, calculation, learning ability, language, and judgment; among the cognitive deficits reported are memory impairment, aphasia, apraxia, agnosia and executive functioning disorders [7,8]. According to these features, the diagnosis of dementia or major neurocognitive disorder based on the Diagnostic and Statistical Manual of Mental Disorders, Fifth Edition, (DSM-5) requires the presence of substantial impairment in one or (usually) more cognitive domains, severe enough to interfere with independence in daily activities [8,9]. Parallel with cognitive decline, which has always been considered the clinical hallmark of AD, some 99% of patients experience fluctuating neuropsychiatric symptoms (NPSs) during the course of the disease [10], termed behavioral and psychological symptoms of dementia (BPSD), the treatment of which remains the most difficult long-term challenge [11]. The definition of these symptoms is very complex, due to their similarity to other symptoms belonging to different neuropsychiatric conditions and they are highly disabling, significantly reducing the quality of life of patients [12]. According to the Cache County Study, patients suffering from dementia experience at least one of these fluctuating symptoms as the disease evolves [13]. In particular, agitation is one of the most difficult symptoms to treat and it is responsible for a remarkable reduction in quality of life due to its main characteristics, which consist of verbal and/or physical aggression and excessive motor activity [14]. States of agitation represent a very serious problem, as they make patients dangerous for themselves and others [15]. States of agitation worsen as the pathology progresses into severe dementia, accompanying patients throughout their lives [16]. Agitation is a predictive factor for a negative prognosis and an increased risk of accidents, responsible for comorbidities and often fatal falls; growing evidence suggests that these symptoms do not depend exclusively on cognitive deterioration but on peculiar dysfunctions of the neurotransmitter systems that are not yet well known [17,18].

Health Technology Assessment (HTA) is a section of health research that aims to generate information about the cost-effectiveness of health technologies. In particular, it is “a multidisciplinary process that summarizes information about the medical, social, economic, and ethical issues related to the use of a health technology aimed to inform the formulation of safe, effective, health policies that are patient-focused and seek to achieve the best value” [19]. According to the World Health Organization (WHO), the term “Health Technology” includes clinical technologies (medical procedures, medical devices, drugs, and medical materials); support technologies (infrastructure and hospital systems, energy systems, information systems and communication, and the organization itself); community health technologies for prevention, protection, and promotion; technologies for environmental health [20]. Thus, health technology refers to medicinal products; medical devices; medical and surgical procedures; public health programs and support systems; organizational and managerial systems for prevention, screening, diagnosis, treatment, and rehabilitation; and equipment. Healthcare represents one of the world’s largest and fastest-growing industries; the industry is significantly impacting the economies and gross domestic product of developed countries. Further in-depth discussion highlights the critical role of the medical device industry in the advancement of healthcare technology and patient-related outcomes [21].

NanoBEO is an innovative device that uses nanotechnology to release bergamot essential oil (BEO) deprived of bergapten, guaranteeing the absence of phototoxicity [22]. The formulation is contained in an airless dispenser, which guarantees the administration of a known dose of the formulation and has the appearance of a cream. The dosage is extremely simple as it is sufficient to administer the product to the patient’s two forearms and massage for a few minutes. The nanotechnological structure allows for the elimination of the odor and volatility of the compounds. The preclinical studies carried out so far have demonstrated the ability of BEO to modulate neurotransmitter levels at the synaptic level in the hippocampus, suggesting the existence of effects on behavior and pain. Furthermore, the absence of aroma allows for the generation of solid double-blind evidence for clinical studies and avoids the possibility of an adverse effect, especially in dysosmic patients (a fairly common situation in AD patients). The anxiolytic effect of BEO is also known. The solution provided by NanoBEO offers the first needs-oriented treatment for agitation in patients with severe AD, which can be administered by all caregivers in their own homes and assisted healthcare residences; it is safe for elderly patients, even those undergoing multiple treatments, and those in paraphysiological conditions. It is effective and usable over the long term. Based on the WHO definition, a health technology assessment (HTA) is a systematic and multidisciplinary evaluation of the properties of health technologies and interventions covering both their direct and indirect consequences. The significance of the unmet medical need originating from treating agitation during the course of dementia warrants conducting HTA research to highlight the role of NanoBEO. The present research focuses on the clinical and market analysis aimed at the HTA of NanoBEO. HTA for NanoBEO aims to provide the systematic evaluation of properties, effects, and impacts of the health technology NanoBEO, addressing its direct, intended consequences, as well as its indirect, unintended consequences. Therefore, the purpose of the present study is the production of the HTA-like report for NanoBEO in dementia, accounting for its effectiveness, cost-effectiveness, and economic, social, and organizational impacts.

## 2. Materials and Methods

### 2.1. Panel Assessments

The main purpose of HTA is to inform technology-related policymaking in healthcare, providing the so-called bridge between evidence and policymaking. Therefore, the HTA of NanoBEO was conducted by an interdisciplinary HTA panel using an explicit analytical framework. This framework relies on the integrative methods of existing systematic reviews, meta-analyses, and preclinical and clinical studies to formulate findings.

To assess the technology, the HTA panel gathered evidence addressing the following issues:

- The burden of the considered disease;
- Projected epidemiological trends of the disease;
- The relative effectiveness, added clinical benefits, and cost-effectiveness of existing health technologies through a systematic approach;
- The direct and indirect impacts of the technology through relevant measures of impact;
- How stakeholders and patients value the investigated health technology;
- Intellectual property assets for commercialization;
- Financial highlights and business plan.

### 2.2. Database Screening

Producing preclinical rodent model data and clinical results demonstrating the efficacy and safety of NanoBEO was accompanied by database screening, selecting results, and extracting data about the clinical benefits and cost-effectiveness of existing health technologies; this process was performed in agreement with the Preferred Reporting Items for Systematic reviews and Meta-Analyses (PRISMA) 2020 recommendations [23,24]. The research questions were formulated using the participants/population, interventions, comparisons, outcomes, and study design (PICOS) framework. Specifically, participants were patients affected by AD; intervention was represented by competitors of NanoBEO, i.e., previous health technologies used for treating agitation in dementia rather than no treatment (comparison); outcomes included a measurable reduction in agitation; and studies deemed to be eligible were meta-analyses aimed at addressing this question. Studies not available in full text in English were excluded. PubMed/MEDLINE, Scopus, and Web of Science were searched for studies published from database inception to the date of the last search on 14 June 2024. The search was performed by two independent members of the panel and the process of removal of duplicate records was carried out using reference manager software (EndNote X7, Clarivate, London, UK).

### 2.3. Search Strategy

For the assessment of treatments and rehabilitation approaches available, the following medical and subject heading (MeSH) terms and subheadings were used for PubMed/MEDLINE searching: (“Alzheimer Disease” [Mesh]) AND “Behavioral Symptoms/rehabilitation” [Mesh]; Scopus was searched using Article Titles, Abstracts, and Keywords, and Web of Science was searched for all fields. The search was conducted with a high sensitivity/recall search strategy [25]. Two requestors independently searched the databases, while a third member checked the accuracy and efficiency of strings in agreement with evidence-based guidelines for Peer Review of Electronic Search Strategies (PRESS) for systematic reviews (SRs) [25,26]. Any disagreements were to be resolved by consensus or consultation with a third team member.

### 2.4. Business Plan Production

The production of the business model canvas and corresponding business plan was based on the main phases of the innovation process, with implications for the firm’s trade policy from ideation and development through to deployment [27]. Intellectual property and patenting were considered, with NanoBEO being a health technology endowed with disruptive innovation. Market choices were analyzed, among mass, niche, segmented, diversified, and multi-sided approaches [28]. The NanoBEO case was investigated through iterative phases to answer open-ended questions. In-depth, semi-structured interviews and focus groups with stakeholders, physicians, neurologists, geriatricians, neuropsychologists, and associations of patients were conducted about the retrospective perspective and to test the feasibility of the developed framework [29]. Direct and indirect market analyses were performed. The best-suited technology transfer models identified among the nine most common for health technologies are as follows [30]: open innovation, sustainable, dynamic, dual, spin-off, frugal, high-tech entrepreneurial content marketing, back-end, and product-service systems.

### 2.5. Academic Spin-Off

**Figure 1:**
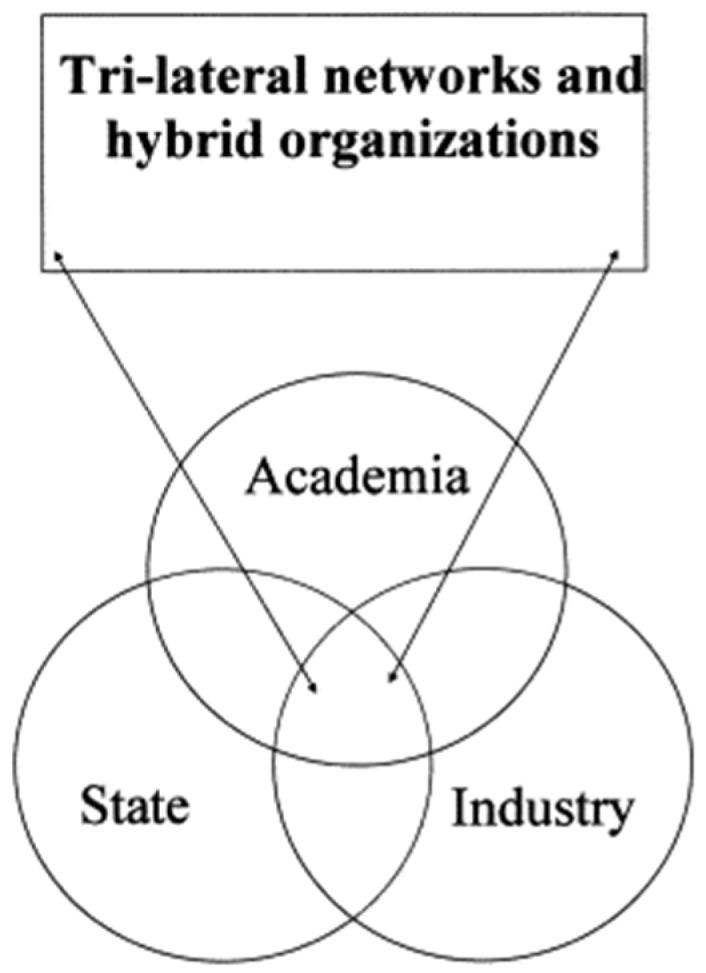
The Triple Helix Model III of university–industry–government relations. This is the spin-off model chosen for NanoBEO. Reproduced with permission from [33].

The operational strategy of the academic spin-off was selected. An academic spin-off is defined as part of an organization (the university research group), which becomes an independent unit, using the assets, intellectual property, employees, and existing products of the parent company [31]. The innovation-driven strategies and operational practices needed for the success and sustainability of the spin-off based on the health technology NanoBEO were investigated. Particular attention was paid to the fundamental role of academic spin-offs in the valorization of research results and commercialization of ideas overcoming the so-called Death Valley. Since university research groups mainly focus on basic research and are devoid of the effective commercial apparatus for the research and development (R&D) of products, an ideal triple helix (university–industry–government) III model for knowledge-intensive network transition was adopted [32,33]. In fact, in the first triple helix model (I), the spin-off is largely independent and separate from the parent institution. In the “laissez-faire” triple helix II model, it migrates from the academic sphere to the industry [32]. On the contrary, in the triple helix III model chosen for the HTA of NanoBEO, the spin-off society is a knowledge infrastructure with overlapping institutional spheres and hybrid interchanging roles [32] (Figure 1). In this specific case, the spin-off represents a bridge between academia (i.e., its parent institution) and industry in need of incentives and funds aimed at technology transfer from national systems and government institutions.

### 2.6. Data Analysis

The data obtained were analyzed and represented as tabular results and the operational models were generated using Microsoft Office Excel 2010. The value chain analysis was conducted using the Online Value Chain Analysis Tool Visual Paradigm Online (https://online.visual-paradigm.com/diagrams/features/value-chain-analysis-tool/; accessed on 14 August 2024).

## 3. Results

### 3.1. Problem and State-of-the-Art Assessment

One in five women and one in ten men are at risk of developing dementia, with an overall prevalence of 10.9%; AD represents the fifth leading cause of death in people aged over 65 years, accounting for its global burden [34]. Incidentally, 74% of caregivers report concerns about maintaining their own health since becoming a caregiver [34]. Projections for health and long-term care costs for people living with dementia are estimated to reach USD 360 billion in 2024 and nearly USD 1 trillion in 2050 [34].

The occurrence of BPSD is characterized by three main phases, detectable as (1) irritability and depression; (2) agitation and anxiety; and (3) psychotic symptoms, such as hallucinations and motor alterations. These symptoms are highly disabling [12], and are experienced in a fluctuating manner as the disease evolves [13]. In particular, states of agitation worsen as the disease progresses into severe dementia, accompanying patients throughout their lives [16]. Agitation is a negative prognostic factor of the increased risk of accidents and it is responsible for comorbidities and often fatal falls. The pathogenesis of AD is explained by the amyloid hypothesis, formulated in the 1980s, according to which the deposition and accumulation of β-amyloid (Aβ) monomers into oligomers, protofibrils, fibrils, and plaques responsible for neurodegeneration [35,36]. Thus, targeting Aβ is supposed to produce clinical benefits [37]. Other pathognomonic hallmarks of AD are the neurofibrillary tangles (NFTs) containing bundles of paired helical filaments of the hyperphosphorylated microtubule-associated tau protein [38,39,40]. The drugs available for the pharmacological treatment of AD are acetylcholinesterase inhibitors (AChEIs), such as donepezil, rivastigmine, and galantamine. A drug more effective in the stages of moderate/severe cognitive impairment induced by AD is the non-competitive N-methyl-D-aspartate-glutamate receptor antagonist, memantine. However, this drug has a very controversial effect on agitation as it exerts modest efficacy in delaying the onset, but it does not affect existing agitation [41]. New strategies targeting Aβ were investigated to delay the progression of AD, but both small molecules and immunotherapy failed. In particular, there are several ongoing clinical trials assessing the effects of disease-modifying therapies (DMTs), including small molecules such as simufilam [42] (NCT05575076; NCT04994483), and the most novel passive immunotherapies, like remternetug, directed toward a pyroglutamated form of Aβ (NCT05463731); all these studies highlight that the clinical target is represented by the amnestic mild cognitive impairment (aMCI) and prodromal AD stages [43]. The mechanisms range from neuroprotection, anti-inflammation, and cognitive enhancement to neuropsychiatric control and autophagy induction to reduce neurodegeneration [44,45,46]. The biotech revolution has not yet managed to provide a cure for AD. Bapineuzumab, a humanized anti-Aβ mAb, was tested in two double-blind, randomized, placebo-controlled phase III studies in mild to moderate AD, demonstrating no improvement in established clinical endpoints (Janssen Alzheimer Immunotherapy and Pfizer; NCT00575055, NCT00574132, and EudraCT number 2009-012748-17) [47]. Also, the two-phase III, multicenter, placebo-controlled, double-blind EXPEDITION 1 and EXPEDITION 2 studies in mild to moderate AD demonstrated that solanezumab, a humanized monoclonal antibody (mAb) that binds soluble Aβ, did not achieve significant results on primary endpoints and failed to improve cognitive status and functional ability (Eli Lilly, NCT00905372 and NCT00904683) [48]. No mAbs of the first (toward aggregated plaques), second (toward soluble Aβ), or third generation (toward oligomeric and fibrillar aggregates) were found to represent the definitive therapy for AD, despite the fast-track approval phase of aducanumab (EMERGE and ENGAGE clinical trials) [49]. On 6 June 2023, the FDA converted the accelerated approval of lecanemab (Leqembi; lecanemab-irmb) to mainstream approval following the CLARITY trial (NCT03887455) [50]. Lecanemab is the first anti-Aβ mAb to be converted from accelerated approval to traditional approval for the treatment of early AD [51]. However, lecanemab is only indicated in the initial and mild stages of the disease and its appropriate use recommendations, as evidenced by the clinical trial [52]. In fact, adverse events related to lecanemab include amyloid-related imaging abnormalities (ARIAs) with edema (ARIA-E) or hemosiderin deposition (ARIA-H), along with microhemorrhages and rare macro-hemorrhages [50]. Since the concurrent use of anticoagulant drugs increases the risk of hemorrhage, the AUR recommends that patients treated with anticoagulants not receive lecanemab [52]. Moreover, patients who are apolipoprotein E ε4 (APOE4) gene carriers, especially those who are homozygotes, are at higher risk for AD development, but also for ARIA during treatment with lecanemab [50], and the AUR recommends APOE genotyping to identify patients who are candidates to receive lecanemab [52]. Also, in July 2024, donanemab (Kisunla; donanemab-azbt) was approved by the FDA for the treatment of early AD, as it has been shown to slow cognitive and functional decline, representing the first and only amyloid plaque-targeting therapy based on a limited-duration treatment regimen according to amyloid plaque removal [53].

Therefore, the DMT under investigation and the sole mAbs approved are aimed at earlier phases of the disease, neglecting the moderate-to-severe stages in which agitation becomes more challenging, and can be used by a narrow segment of AD patients due to comorbidities. Indeed, the majority of patients suffering from AD are over the age of 65; this group often suffers from comorbidities that increase the risk of cardio-cerebrovascular accidents, predisposing them to anticoagulant therapy. This is a matter of the utmost importance for agitation therapy. Its treatment relies on risperidone, an atypical antipsychotic. On 11 May 2023, the FDA announced the supplemental approval of brexpiprazole [54]. However, these drugs potentially increase the risk of death from cardiocerebrovascular accidents [54,55]. In fact, risperidone cannot be used for longer than 6–12 weeks because it increases the risk of death from heart infarction, stroke, and serious infections by a factor of 1.6 to 1.7. Other drugs tested include antidepressants (e.g., tricyclic antidepressants, trazodone, venlafaxine, and selective serotonin reuptake inhibitors (SSRIs), such as sertraline and, more recently, citalopram and escitalopram); anticonvulsants and mood stabilizers (e.g., carbamazepine, gabapentin, valproic acid); and benzodiazepines, such as lorazepam. Due to the involvement of multiple neurotransmitter systems in dementia-related agitation, a new experimental drug, lumateperone tosylate, is being studied because it has several potentially useful pharmacological mechanisms [56]. Among these, the most effective proved to be selective serotonin reuptake inhibitors (SSRIs), which, nevertheless, increase the QT interval with possible cardiotoxicity and can also worsen agitation. In particular, QT interval prolongation can lead to the onset of a serious, potentially fatal, ventricular arrhythmia, the torsade de pointes, which can result in sudden death from cardiac arrest. Furthermore, most of these drugs are contraindicated in AD patients because they worsen cognitive decline, leading to an increased risk of falls, institutionalization, and death. Cannabinoids are being investigated as a means to treat agitation during dementia (NCT02351882, EudraCT2020-001056-17 STAND, ACTRN12619000474156 [57,58,59,60,61,62,63,64]). Our research group is working on a double-blind, placebo-controlled, randomized trial (NAbiximols Clinical Translation To the Treatment of Pain and Agitation in Severe Dementia [NACTOPAISD]) to evaluate the efficacy and safety of the oral spray known as nabiximols, which contains Δ9-tetrahydrocannabinol and cannabidiol (Sativex^®^); this trial focuses on the treatment of pain and agitation in patients with severe dementia [64]. It is necessary to keep in mind that cannabinoids are contraindicated in patients suffering from cardiovascular diseases, which are very common in over 65 people. Furthermore, as patients with dementia frequently present age-related comorbidities and are often poly-treated [65], they are at a higher risk due to physiological differences, variability in response to medications [66], drug-to-drug interactions [67], and the lack of inclusion of these patients in clinical trials for pain management and other concurrent diseases [68,69,70,71,72,73]. For all these reasons, the most favorable approaches for the management of BPSD or NPS are represented by non-pharmacological treatments that aim to take care of patients’ needs (needs-oriented), such as music listening and bright light therapy [11].

### 3.2. Evaluation of Non-Pharmacological Treatments for Agitation in Dementia

Aromatherapy, a specialized form of herbal medicine that uses essential oils for inhalation or massage, is a treatment that provides preliminary evidence of effectiveness [74]. Nevertheless, the lack of dose standardization and the effects of the essential oils administered limit their value and rational use in clinics [75]. In particular, aromatherapy with melissa and lavender essential oils proved effective in the management of agitation in dementia [11], but suffers from some weaknesses (methodological biases), limiting its rational use. In fact, aromatherapy does not allow for the administration of a known, stable, and constant dose of the phytocomplex to the patient over the long term, due to the physical–chemical instability of essential oils. Therefore, the effects are not reproducible. Furthermore, the strong aromas of essential oils hinder the adequate masking of their administration for double-blind clinical trials, reducing the quality of the evidence produced by the clinical trials conducted so far, which are also unable to guarantee statistical significance [75]. To acquire complete evidence about the treatments for BPSD rehabilitation, a systematic search was performed, screening PubMed/MEDLINE, Scopus, and Web of Science, from their inception up until 17 June 2024, i.e., the date of the last search, according to PRISMA recommendations. The systematic search retrieved 436 total records, among which, 20 were obtained from PubMed/MEDLINE, 119 from Scopus, and 297 from Web of Science. Forty duplicate records were found. The assessment of the 396 records against the inclusion criteria, which focused on non-pharmacological treatments for BPSD, resulted in 4 systematic reviews to examine. In fact, among the studies that could apparently be deemed eligible, the study by Clay et al., 2020 [76] focused on the detection and cognitive training of AD through immersive virtual reality, thus it was excluded. Also, the study by Rachel et al., 2022 [77] did not meet the inclusion criteria, being an umbrella review reporting the effect of active rehabilitation on tauopathies. The paper by Spira et al., 2006 [78] had to be excluded since it was an evaluative review. The paper by Cross et al., 2022 was not open access and the full text could not be obtained; thus, it was excluded [79]. The study by Bennett et al., 2018 [80] investigated the effect of occupational therapy on activities of daily living (ADL), BPSD, and the quality of life of people with dementia. It detected 15 trials that included patients having a medical diagnosis of mostly moderate-stage dementia with MMSE scores between 11.3 and 22.73. The results showed improvement in ADL with very low to moderate quality evidence assessed using the Grading of Recommendations, Assessment, Development, and Evaluation (GRADE) criteria [81]. The effect of occupational therapy on BPSD was small to moderate with a statistically significant between-group difference in the number of BPSD symptoms and high heterogeneity (I^2^ = 58%) among the six studies selected, likely reflecting the application of different intervention programs in the included studies [80]. The results of studies on anxiety did not show significant improvement and those on depression were controversial. The data combined from three studies yielded no significant differences between occupational therapy and usual care [80]. The study by de Almeida et al., 2020 [82] identified 16 randomized, controlled trials, most of them of high quality, but with large heterogeneity of interventions, including patients with an MMSE mean score of 19.9 ± 5.9 [15.3, 25.6] (hence, participants were not in severe stages of dementia). The study investigated the effects of home-based physical activity on cognitive and behavioral activity levels and quality of life domains. Six studies evaluated the neuropsychiatric inventory score (NPI). A small improvement in BPSD was reported, but no significant differences were highlighted in the Cornell Scale for Depression in Dementia, Short Physical Performance Battery, and Dementia Quality of Life [82]. The meta-analysis for NPI demonstrated small effect sizes with high heterogeneity (I^2^ = 97%) [82]. The study by Wang et al., 2018 [83] reported sixty-four clinical trials, forty-one of which showed that nonpharmacological interventions afforded significant reduction in at least one BPSD, but the quality of evidence of the trials was low [based on the National Health and Medical Research Council (NHMRC) evidence hierarchy] due to considerations such as lack of capability (i.e., consideration of the capability of people with dementia), data inhomogeneity, and inadequate study design and reporting.

**Figure 2:**
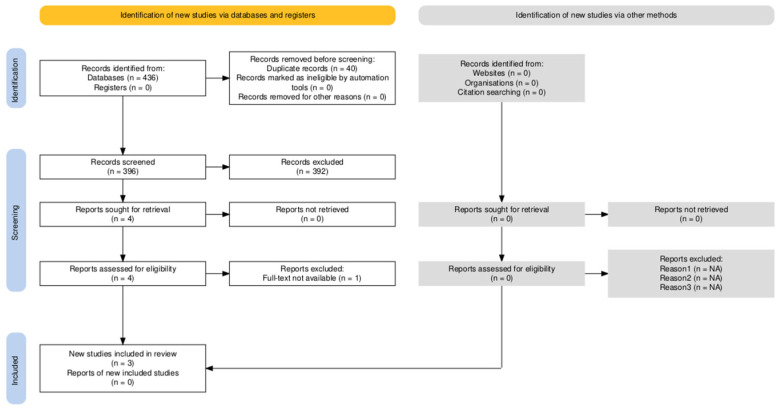
Systematic search and selection of records based on the Preferred Reporting Items for Systematic Reviews and Meta-Analyses (PRISMA) 2020 flow diagram, created using the web-based Shiny app [84].

The selection of the eligible studies is illustrated in Figure 2.

### 3.3. Solution and Value Proposition

**Figure 3:**
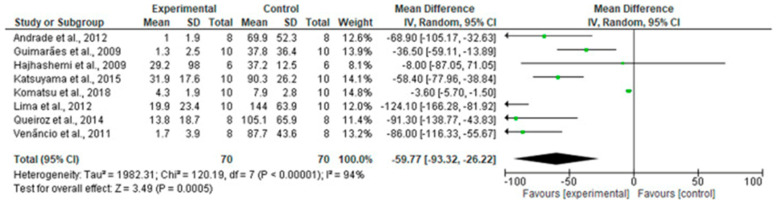
Forest plot for the analgesia provided by essential oils. The results of the meta-analysis favor efficacy with respect to control, but they are affected by high heterogeneity (mean difference MD −59.77; 95% CI (−93.32)–(−26.22); I^2^ = 94%; *p* < 0.00001). Reproduced with permission from [88].

Most preclinical studies on essential oils do not justify their clinical use for treating agitation in dementia, except for bergamot essential oil (BEO, *Citrus bergamia* Risso et Poiteau). In particular, BEO is composed of a volatile (93–96% of total) containing monoterpene and sesquiterpene hydrocarbons, such as limonene, and oxygenated derivatives, such as linalool and linalyl acetate. BEO comprises a non-volatile fraction (4–7% of total), containing waxes, polymethoxylated flavones, coumarins, and psoralens. Cerebral microdialysis experiments, together with studies performed on synaptosomes, used for the morphological and functional study of synapses and the pharmacological study of neurotransmitters, demonstrated that BEO is involved in the modulation of the levels of synaptic neurotransmitters, such as glutamate, in the hippocampus, suggesting the existence of effects on behavior and pain; the latter mechanism can be significantly implicated in the disorders occurring during aging, like pain, and BPSD development, such as agitation [85]. In fact, group I, (mGluR1,5), group II (mGluR2,3), and group III (mGluR4,6,7,8) metabotropic glutamate receptors can be responsible for central sensitization and neuroplastic modifications fostering chronic pain. BEO also documented effects on autophagy, a process that ensures the cell energy supply by eliminating potentially harmful waste dysregulated in aging and dementia [86]. Furthermore, BEO has an anxiolytic effect [87], which is the basis of its aromatherapeutic use. BEO provided strong preclinical evidence of efficacy in clinically relevant models [88]. This evidence was generated using the PRISMA methodology. The systematic search was conducted to answer the PICOS question, asking if essential oils are effective in the reduction of acute nociceptive pain and/or neuropathic pain in mice experimental models [88]. After retrieving 2491 records and removing duplicates, 954 studies were screened (the title and abstract) for eligibility, assessing the antinociceptive effects of essential oils, with a known percentage of components administered via intraperitoneal or subcutaneous routes to allow determining the exact dose and reproducibility (male mice were subjected to nociceptive or neuropathic pain models in compliance with animal welfare regulations). Of the 127 records evaluated in full text, 27 studies focused on the analgesic properties of essential oils in acute nociceptive pain models, which are not as suitable for human conditions of chronic pain, such as neuropathic pain models and models with central sensitization, which were examined in only 3 studies on BEO. Furthermore, some methods raised concerns in terms of the risk of bias; the meta-analysis of comparable studies favored the analgesic efficacy of essential oils (mean difference, MD −59.77; 95% confidence interval, CI (−93.32)–(−26.22); *p* < 0.00001; Figure 3), but with the need for caution due to the extremely high heterogeneity (I^2^ = 94%) [88]. The sole BEO met all these important criteria and was investigated following the most rigorous methodologies for preclinical testing, such as the guidelines for Animal Research: Reporting In Vivo Experiments (ARRIVE).

**Figure 4:**
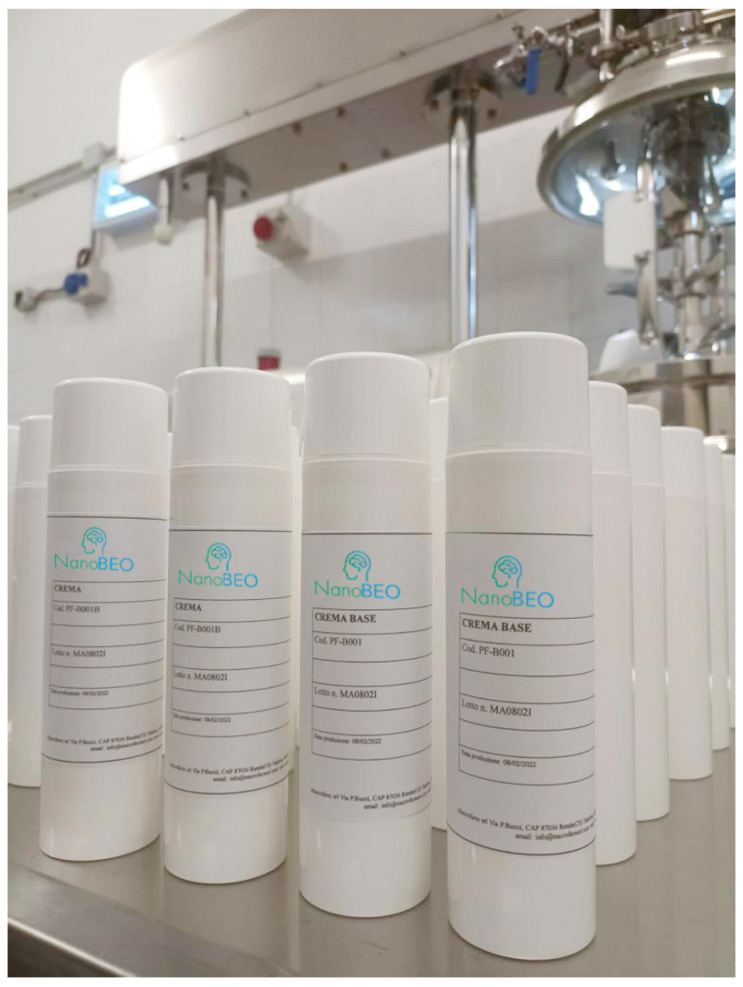
Dispensers of the first batches of NanoBEO.

Cerebral microdialysis experiments, together with studies performed on synaptosomes, used for the morphological and functional study of synapses and the pharmacological study of neurotransmitters, demonstrated that BEO is involved in the modulation of the levels of synaptic neurotransmitters, such as glutamate, and in the hippocampus, suggesting the existence of effects on behavior and pain; the latter mechanism can be significantly implicated in the disorders occurring during aging, like pain, and BPSD development, such as agitation [85]. BEO also documented effects on autophagy, a process that ensures the cell energy supply by eliminating potentially harmful waste, dysregulated in aging and dementia [86]. Furthermore, BEO has an anxiolytic effect [87], which is the basis of its aromatherapeutic use. The fundamental problem of aromatherapy is represented by the impossibility of guaranteeing the exposure of individuals to a known quantity of the phytocomplex, titrated in the main components. In this way, the reproducibility of the observed effects is lost. Furthermore, some volatile components of essential oils are relatively unstable to heat and light. Therefore, BEO was engineered to provide a delivery system to overcome all the limitations of aromatherapy, since the aroma is entrapped, allowing a double-blind clinical trial. In particular, NanoBEO consists of a nanotechnological delivery system based on solid lipid nanoparticles loaded with bergapten-free BEO, guaranteeing the absence of phototoxicity [22]. Moreover, NanoBEO is designed for extreme ease of application (e.g., one supply for each forearm of the patient corresponds to the daily dose). To promote the stability of the formulation and ease of application, the cream is provided in an airless dispenser [22] (Figure 4). The absence of aroma allows for the generation of solid double-blind evidence and avoids the possibility of an aversive reaction, especially in dysosmic patients since anosmia is often one of the first warning signs of the development of AD.

**Figure 5:**
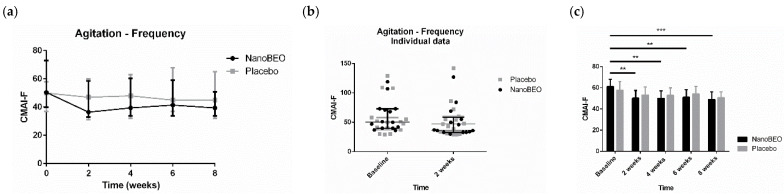
Efficacy of NanoBEO on the frequency of agitation according to the Cohen-Mansfield Agitation Inventory (CMAI-F) during the treatment and follow-up (**a**) and as individual data at baseline and after two weeks of treatment (**b**); data are expressed as median + interquartile range (IQR). Patients allocated to the NanoBEO group experienced a reduction in CMAI-F scores compared to patients allocated to the placebo group; (**c**) the effectiveness of NanoBEO showed statistically significant differences for all the time points versus baseline (data are expressed as mean ± SEM; time factor **** *p* < 0.0001; subjects matching **** *p* < 0.0001; NanoBEO: 2, 4 and 6 weeks ** *p* < 0.05, 8 weeks *** *p* < 0.001). * *p* values < 0.05 are considered statistically significant. n: NanoBEO = 14, placebo = 16. (Reproduced with permission from [94]).

**Figure 6:**
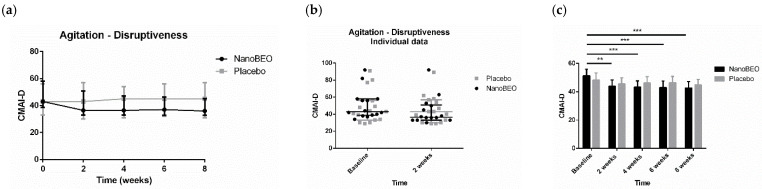
Efficacy of NanoBEO on the disruptiveness of agitation according to the Cohen-Mansfield Agitation Inventory (CMAI-D) during treatment and follow-up (**a**) and as individual data at baseline and after two weeks of treatment (**b**); data are expressed as median + interquartile range (IQR). Patients allocated to the NanoBEO group experienced a reduction in CMAI-D scores compared to patients allocated to the placebo group; (**c**) the effectiveness of NanoBEO showed statistically significant differences for all the time points versus baseline (data are expressed as mean ± SEM; time factor **** *p* < 0.0001; subjects matching **** *p* < 0.0001; NanoBEO: 2 weeks ** *p* < 0.01; 4, 6 and 8 weeks *** *p* < 0.001). * *p* values < 0.05 are considered statistically significant. n: NanoBEO = 14, placebo = 16. (Reproduced with permission from [94]).

**Figure 7:**
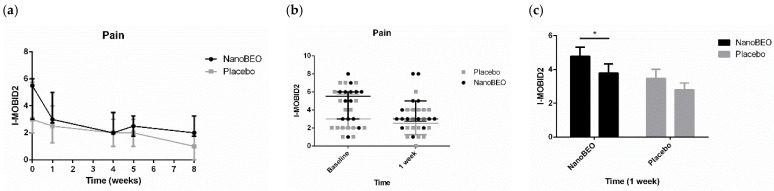
Efficacy of NanoBEO on pain according to Italian Mobilization–Observation–Behavior–Intensity–Dementia (I-MOBID2) during the course of treatment and follow-up (**a**) and as individual data at baseline and after one week of treatment (**b**); data are expressed as median + interquartile range (IQR). Patients allocated to the NanoBEO group experienced a reduction in I-MOBID2 scores compared to patients allocated to the placebo group after one week. The reduction continued up until the end of treatment and increased during the follow-up period, without reaching the baseline value; (**c**) the effectiveness of NanoBEO proved statistically significant (data are expressed as mean ± SEM; time factor ** *p* = 0.0031; Subjects matching **** *p* < 0.0001; NanoBEO baseline vs. 1 week * *p* < 0.05). * *p* values < 0.05 are considered statistically significant. n: NanoBEO = 14, placebo = 16. (Reproduced with permission from [94]).

NanoBEO demonstrated preclinical efficacy in models, paralleling the typical clinical conditions of the elderly and patients suffering from severe AD. The solid nano-lipid nanoparticles protect the BEO from chemical–physical instability, ensuring a high shelf-life. In particular, the results of the preclinical studies using NanoBEO demonstrated stable and reproducible aromatherapeutic effects: after two and six months of exposure to light, the contents of active ingredients decreased by 10% and 18% respectively, without any further degradation at twelve months [22]. As BEO, NanoBEO proved strong efficacy over control in the capsaicin inflammatory pain models, in the formalin test, and the partial sciatic nerve ligation (PSNL) neuropathic pain model [89]. Furthermore, NanoBEO was also effective in preventing 4-methyl-histamine-induced scratching behavior, which is also a typical BPSD [89]. The control consists of the cream incorporating empty solid lipid nanoparticles. Also, the pharmacokinetic profile of the volatile constituents of BEO released after transdermal administration of NanoBEO (D-limonene, linalool, and linalyl acetate) was determined through an accurate gas chromatography–mass spectroscopy on plasma samples. NanoBEO allows for easy and simple cutaneous administration of a certain and stable dose that is effective and safe for treating agitation during dementia. In fact, preclinical research established a dosage in accordance with FDA guidelines for converting effective and safe doses from preclinical to clinical trials [90], which was used in the pilot phase of the trial BRAINAID (NCT04321889) [91]. The trial was approved by the Calabria Region Ethics Committee, protocol no. 352 first version (21 November 2019), and followed the Standard Protocol Items: Recommendations for Interventional Trials (SPIRIT) [92] and the Consolidated Standards of Reporting Trials (CONSORT) statements [93]. In this randomized, double-blind, placebo-controlled trial with quadruple masking of all the operators and participants, 29 patients aged ≥ 65 years suffering from severe dementia were randomly allocated to the NanoBEO or placebo group in a 1:1 allocation ratio, and NanoBEO was effective in both on agitation and pain, in a statistically significant manner, with a clinically meaningful percentage of reduction (Figure 5, Figure 6, Figure 7; reproduced with permission from [94]). In particular, patients were administered one supply containing a dose of 1 g of active cream or a placebo cream on each arm once a day for 4 weeks, so that one dispenser covered the whole 4-week-treatment. The patients receiving NanoBEO showed both reduced frequency and disruptiveness of agitated behaviors, in comparison to patients allocated to the placebo group in a statistically significant manner. The improvement in the frequency of agitation reached 28% in the NanoBEO group, in comparison with 6.93% in the placebo group [94], almost up to the 30% threshold generally regarded as clinically significant in trials for BPSD treatment [95]. The efficacy lasted for the whole 4-week treatment period, decreasing during the follow-up for frequency. The reduction in pain intensity after the first week of treatment reached 45.46% in comparison with 16.67% for the placebo [94], hence exceeding the threshold of 30% improvement generally regarded as clinically important in trials on pain, according to the Initiative on Methods, Measurement, and Pain Assessment in Clinical Trials (IMMPACT) recommendations [96]. NanoBEO was well-tolerated, with no patients discontinuing the trial due to adverse reactions related to the treatment; moreover, no side effects were reported at any of the 4-week assessments or during the follow-up. Moreover, the biochemical analyses were not influenced by the treatment with NanoBEO [94].

The main competitors of NanoBEO are risperidone, off-label psychotropic drugs, aromatherapy, and other non-pharmacological therapies that are classified as sensory-oriented, cognition-oriented, and movement-oriented [83]. NanoBEO is the first treatment for agitation in patients suffering from severe dementia; it is needs-oriented, safe, effective, and designed for long-term administration, as highlighted in Table 1. These data are confirmed by the Resource Utilization in Dementia (RUD) instrument, which is an assessment tool developed and validated to evaluate the use and cost of economic resources for the management of patients with dementia [97]. NanoBEO, in addition, works earlier than the novel brexpiprazole [98] and it is safer compared to citalopram [99].

### 3.4. Intellectual Property

On 30 July 2019, the patent filing for NanoBEO was filed by the University of Calabria, which was granted on 21 July 2021 (concession number 102019000013353). The patent inventors are as follows: Bagetta Giacinto (Italy), Cassano Roberta (Italy), Trombino Sonia (Italy), Russo Rossella (Italy), Mizoguchi Hirokatsu (Japan), Watanabe Chizuko (Japan), Hamamura Kengo (Japan), Katsuyama Soh (Japan), Komatsu Takaaki (Japan), Morrone Luigi Antonio (Italy), Rombolà Laura (Italy), Adornetto Annagrazia (Italy), Lagana Annarita Stella (Italy), Scuteri Damiana (Italy), Corasaniti Maria Tiziana (Italy), Tonin Paolo (Italy), Sakurada Shinobu (Japan), Sakurada Tsukasa (Japan), and Nicotera Pierluigi (Germany). The European patent filing, for which the Sant’Anna Institute and the German Center for Neurodegenerative Diseases (DZNE) are also patent holders, along with the University of Calabria, was published on 1 June 2022 with the official number EP 4003294.

### 3.5. Macro-Market Analysis

According to the report “Dementia Drugs Market Research, 2031”, the value of the market for drugs for the treatment of dementia in 2021 was USD 8.7 billion, and it is projected to reach USD 19.7 billion in 2031 at a compounded average growth rate (CAGR) of 8.5% (2022–2031) [100]. Among the diseases responsible for the cognitive deterioration of patients are Lewy body dementia, Parkinson’s dementia, AD, vascular dementia, and other diseases with a lower incidence. However, AD accounts for approximately 2/3 of cases, therefore, it was decided to focus the analysis on AD only.

The global costs linked to AD amounted to USD 1313.4 billion in 2019, also considering the costs of “Informal care”. The market for drugs for the treatment of AD will reach USD 13.7 billion in 2030, considering only eight countries (France, Germany, Italy, Spain, UK, Japan, and China) with a CAGR of 20% [100]. As is well-known, the incidence of AD increases with age: statistics show that 5% of the population between 65 and 74 years old suffer from this disease; the percentage rises to 13.1% between 75 and 84 years old and reaches 33.3% in the population over 85 [101]. Thus, it is possible to determine a total addressable market (TAM) of over 55 million patients with a strong growth trend if no solutions are found; in fact, it is expected that the population of AD patients will reach 78 million in 2030, and 139 million in 2050. This matter is of the utmost importance, as strengthened already in the early 2000s by the annual indirect cost for management of BPSD in an AD patient of approximately USD 2665, i.e., over 25% of the total annual indirect cost of care (USD 10,520), and by the annual direct cost of approximately USD 1450, i.e., over 35% of the total annual direct cost of care (USD 3900) [102]. The served addressable market (SAM) was quantified in a total of 7,853,705 [103] patients, representing the segment of the population suffering from AD in Europe. The segmentation proposed here is based on direct market surveys conducted with neurologists and healthcare professionals of the Center for Cognitive Disorders and Dementia (CDCD) and general practitioners (GPs) of the relevant healthcare district, as well as with national associations of patients (surveys with pharmacologists, neurologists, and neuropsychiatrists of the DZNE). Possible new target markets may include patients suffering from other neurodegenerative diseases, such as Parkinson’s disease, ischemic or hemorrhagic stroke, and those who develop post-stroke agitation and pain, serving as an add-on therapy to other non-pharmacological approaches [104].

### 3.6. Marketing and Industrial Plan

One dispenser contains the quantity of product needed to treat a patient for a month. As already detailed in the value proposition, NanoBEO offers several advantages, as follows: (1) **Needs-oriented**: It responds to unmet needs, i.e., the unmet needs that represent the main triggers of physical and verbal agitation, considered by the latest international guidelines to be the treatment of choice in these fragile patients [11] who suffer from various comorbidities and paraphysiological conditions accompanied by poly-therapies characterized by a high potential for toxicity. (2) Titration in the dose: It allows the patient to be exposed to a constant and stable dose over time and, therefore, the patient obtains reproducible beneficial effects. (3) Safe treatment: It provides a long-term treatment option that can eliminate or at least limit the use of drugs that are harmful to the patient or worsen their general health conditions, prolonging the “time in therapy”. Other indirect competitors, such as risperidone and aromatherapy, are not authorized or approved for the treatment of psychological and behavioral symptoms of dementia, but are used off-label, presenting comparable costs, and are devoid of the advantages of NanoBEO.

**Figure 8:**
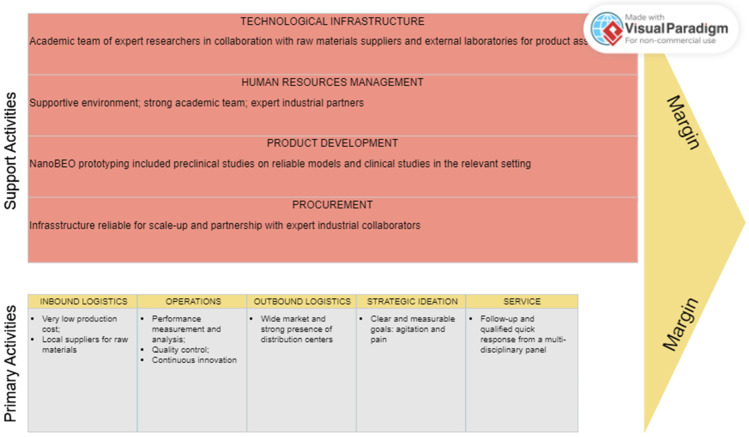
NanoBEO Porter’s value chain analysis.

The values underpinning NanoBEO are as follows: responsiveness to needs; effectiveness; safety; and simplicity of use. The value chain diagram, according to the model theorized by Michael Porter in 1985, reported in Figure 8, shows that most of the primary chain activities (inbound and outbound logistics, production, and service) are already operational and that the sole aspect to be defined relates to marketing and sales. Furthermore, Porter’s support activities, spanning all primary activities, have already been planned to increase the unique competitive advantages of NanoBEO. In particular, for executive management, accounting, legal counsel (firm infrastructure), and human resource management, there are plans to hire new employees, supported by applications for financial grants, as well as plans to create more efficient internal processes. In particular, setting up the technological value chain for an academic spin-off implicates the identification and development of a series of activities that create value for the end customer, i.e., the patient, using technology as a central element. Here are the key steps considered to build this value chain:

- Analysis of the market and unmet medical needs of patients suffering from AD: It was necessary to understand AD patients’ needs and to identify target segments. Furthermore, competitors were studied to identify strengths and weaknesses, offering novel opportunities for differentiation due to patient relief. NanoBEO emerged as the sole treatment oriented toward the needs of AD patients affected by agitation.
- Strategic ideation and planning: Clear, measurable goals were set in order to drive innovation and product development. These targets focus on providing relief from agitation and pain with a treatment feasible for the long term.
- Product development: NanoBEO prototyping included preclinical studies on reliable models and clinical studies in relevant settings for the market launch.
- Technological infrastructure: The most suitable technological tools for NanoBEO development, deployment, and monitoring are provided by the academic teams of expert researchers and via collaboration with raw material suppliers and external laboratories for product assembly. The infrastructure has already proven to be reliable for scale-up, even for a wider market. In addition, collaborations with nursing homes were established.
- Performance measurement and analysis: Systems to monitor key performance indicators and the effectiveness and safety of NanoBEO were obtained through extensive preclinical studies and by the pilot phase of the clinical trial. Moreover, a feedback loop is under investigation to improve the product based on data collected from the real-world setting. KPIs include measuring the efficacy of agitation treatment using the CMAI and assessing pain via the I-MOBID2 assessment, along with the re-evaluation and monitoring of safety and any side effects over time.
- Service to patients: Follow-up and qualified quick responses from a multi-disciplinary panel are guaranteed.
- Continuous innovation: The academic team will be committed to continuous research and development, defining a pipeline to ensure quality and improvements, and forming strategic alliances with other companies or institutions to exploit new technologies and skills that support sustainable growth and continuous innovation. Also, a workflow for collecting, processing, and securing data storage was implemented.

## 4. Discussion

### 4.1. Unmet Need for Novel Health Technologies and Their Assessment in Dementia Management

Agitation is one of the most difficult symptoms to treat and causes a significant reduction in the quality of life of patients who may demonstrate verbal and/or physical aggression and excessive motor activity. States of agitation represent a very serious problem as they make these subjects dangerous to themselves and others. Indeed, BPSD is often under-recognized, and an investigation of Mild Behavioral Impairment [105] has not improved pharmacological therapy [106]. Furthermore, agitation, like all NPSs, worsens as the disease advances, accompanying patients throughout their lives. To date, there are no specific solutions for treating agitation, and neuroleptics cannot be used for longer than 6 weeks due to a 1.6/1.7-fold increased risk of death from cardiocerebrovascular accidents. In order to address this important, unmet medical need, HTA intends to launch NanoBEO to the market; this device consists of a delivery system, based on solid lipid nanoparticles loaded with BEO, deprived of bergapten, guaranteeing the absence of phototoxicity. The formulation is contained in an airless dispenser, which allows for the administration of a known dose of the formulation with the appearance of a cream. The administration is extremely simple as it is sufficient to administer the product on the patient’s two forearms and massage for a few minutes. The nanotechnological structure allows for the elimination of the odor and volatility of the compounds. The preclinical studies carried out so far have demonstrated the ability of BEO to modulate neurotransmitter levels, inducing the exocytosis of glutamate, which modulates pain through mGluRs and is involved in the release of endogenous opioid peptides and endocannabinoids with analgesic activity [5], suggesting the existence of effects on pain and behavior through peripheral mechanisms [107]. The describe–investigate–create–evaluate (DICE) model suggests that disruptions in brain circuitries are predisposing factors that enhance vulnerability to triggers such as pain and BPSD development [108,109,110,111]. Moreover, the Italian Silver Network Home Care project demonstrated that only 25% of the 49% of elderly patients who experience daily pain receive an I-level analgesic [112].

### 4.2. NanoBEO as an Innovative Health Technology

The absence of aroma in NanoBEO facilitated the generation of solid double-blind evidence from the BRAINAID pilot phase clinical trial, demonstrating statistically significant efficacy in reducing agitation (28% reduction) and pain (45% reduction) [94]. Also, it avoids the possibility of an aversive effect, especially in dysosmic patients (a fairly common situation in AD patients). Interestingly, the results obtained in the clinical trial with NanoBEO are even more important, considering the high placebo response rates previously registered [113] and the modest efficacy of neuroleptics [114]. In 2019, the global costs related to AD amounted to USD 1313.4 billion, also considering the costs of “Informal care”. The market for drugs for the treatment of AD alone will reach USD 13.7 billion in 2030, considering only eight countries (France, Germany, Italy, Spain, the UK, Japan, and China) with a CAGR of 20%. It is possible to determine a TAM of over 55 million patients worldwide, with a strong growth trend if no definite solution applicable to all the types of AD patients is found. In fact, the population of patients suffering from AD is estimated to reach 78 million in 2030, and 139 million in 2050. The SAM amounts to 7,853,705 patients, representing the segment of the population suffering from AD in Europe. For the feasibility analyses, 100% of patients suffering from AD were considered as the target population, given an incidence of agitation in 99% of them. An effective and safe treatment for dementia-related agitation remains a challenge. In fact, no direct competitors have emerged, i.e., companies that are developing or are already on the market with products specifically formulated and intended for the purposes for which NanoBEO was created. However, clinical studies are currently underway; moreover, the use of cannabinoid-based formulations would present a potential risk of toxicity in polymorbid and poly-treated patients, such as elderly patients suffering from AD. No evidence for the effectiveness of aromatherapy can be drawn according to the Cochrane systematic review, due to the methodological biases of clinical trials [75,115]. An extrapolation of the effects of antipsychotics in the treatment of primary neuropsychiatric disorders has led to their off-label use in dementia, regardless of the differences between primary and secondary disorders in terms of neuropsychopathology and, consequently, drug efficacy and safety. On the contrary, no side effects were recorded at any of the 4-week assessments or during follow-up in the BRAINAID (NCT04321889) [91] pilot phase. Also, the biochemical analyses (azotemia, serum creatinine, creatine phospho-kinase, and transaminase levels) were not affected by treatment with NanoBEO, proving the safety of the product. Also, pain treatment was burdened by complications. In fact, oral non-steroidal anti-inflammatory drugs used for inflammatory musculoskeletal pain and celecoxib for the treatment of chronic osteoarthrosis, after the failure of acetaminophen, are only recommended for short periods by the American Geriatric Society panel [116,117], due to gastrointestinal, renal, and cardiovascular adverse reactions [118,119,120] (and with caution in case of concurrent use of warfarin) [121]. Tricyclic antidepressants (e.g., amitriptyline) for the treatment of neuropathic pain cannot be used because of their cardiovascular contraindications [122]. Finally, opiates (tramadol, tapentadol, buprenorphine, or transdermal fentanyl) require effective dose titration [123,124], considering liver and/or renal failure, which are common among aged patients. Thus, there are no direct competitors for the proposed solution.

## 5. Conclusions

NanoBEO represents a public-worth innovation in the neglected area of agitation related to dementia, which is of the utmost importance since it worsens over the course of the disease. In fact, a high correlation between the CMAI score and the stage of dementia was found (Spearman rho = 0.421, *p* = 0.000) [125]. Relevant primary preclinical and clinical data studies supporting the use of this device and the production of the operative plan for its launch on the market were provided, offering recommendations for its implementation in dementia. Therefore, NanoBEO is unique since it represents the first needs-oriented treatment, originating from a step-by-step preclinical-to-clinical pathway, proving both preclinically and clinically to be effective, safe, and easy to administer for the long term.

## Figures and Tables

**Table 1:** Competitive advantages of NanoBEO compared to the other available treatments. Arrows up = increased; arrows down = decreased.

	NanoBEO	Cognitive Symptomatic Treatment	Risperidone/ Antipsychotics	Off-Label Psychotropic Drugs	Non-Pharmacologic Treatments and Aromatherapy
Efficacy	*Needs-oriented*, stable titrated dose, feasible administration for a long time	Delay of symptoms	Mechanism of action not specific for dementia-linked agitation. Potentially harmful for increased risk of stroke and heart failure if used longer than 6–12 weeks	Mechanism of action not specific for dementia-linked agitation. Worsening of cognitive decline	Not significant because of methodological biases; uncertainty of the dose
Safety	Devoid of side effects as demonstrated by the pilot phase of the trial BRAINAID (NCT04321889) [European Medicine Agency (EMA), 13 September 2011 EMA/HMPC/56155/2011 Committee on Herbal Medicinal Products (HMPC)]	Cardiac toxicity, hypertension, dizziness, headache, constipation	X 1.6–1.7 risk of death [55]	Increased cognitive impairment and risk of fatal falls	Not adequately documented
Costs for hospitalization	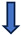	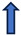	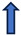	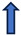	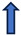

## Data Availability

The original contributions presented in this study are included in the article; further inquiries can be directed to the corresponding author.
